# Leveraging Social Media to Achieve Population-Level Reach of Lung Cancer Screening-Eligible Individuals: A RE-AIM Framework Perspective

**DOI:** 10.2196/80281

**Published:** 2026-03-03

**Authors:** Lisa Carter-Bawa, Jamie S Ostroff, Susan M Rawl, Erin A Hirsch, Smita C Banerjee, Andrew Ciupek, Robert Skipworth Comer, Minal Kale, Katherine T Leopold, Patrick O Monahan, James E Slaven Jr, Francis Valenzona, Renda Soylemez Wiener, Ana Guadalupe Vielma

**Affiliations:** 1 Cancer Prevention Precision Control Institute, Center for Discovery & Innovation, Hackensack Meridian Health, 123 Metro Blvd, 6th Floor, 6400 Pod, Nutley, NJ, 07110, United States, 1 646-246-2118; 2 Cancer Prevention & Control Program, Georgetown Lombardi Comprehensive Cancer Center, Washington, DC, United States; 3 Department of Psychiatry & Behavioral Science, Memorial Sloan Kettering Cancer Center, New York, NY, United States; 4 School of Nursing, Indiana University, Indianapolis, United States; 5 Go2 for Lung Cancer, Washington, United States; 6 School of Informatics, Indiana University, Indianapolis, United States; 7 Department of Medicine, Internal Medicine, Icahn School of Medicine at Mount Sinai, New York, United States; 8 Hackensack Meridian School of Medicine, Hackensack Meridian Health, Nutley, United States; 9 Department of Biostatistics and Health Data Science, Indiana University School of Medicine, Indianapolis, IN, United States; 10 Center for Health Optimization & Implementation Research, VA Boston Healthcare System, Boston, MA, United States; 11 National Center for Lung Cancer Screening, Veterans Health Administration, Washington, DC, United States; 12 The Pulmonary Center, Boston University Chobanian & Avedesian School of Medicine, Boston, MA, United States

**Keywords:** lung cancer screening, RE-AIM, Reach, Effectiveness, Adoption, Implementation, and Maintenance, digital health communication, Facebook-targeted advertisement, implementation science, public health outreach

## Abstract

**Background:**

Annual lung cancer screening (LCS) can decrease lung cancer–related mortality by finding cancer at earlier, more treatable stages, yet uptake remains abysmally low in the United States, especially among adults who seldom interact with the health system. Many eligible individuals are unaware that LCS exists, underscoring the critical need for scalable, population-level communication strategies that increase awareness and engagement.

**Objective:**

The aim of this study was to evaluate *reach*, as defined by the Reach, Effectiveness, Adoption, Implementation, and Maintenance framework, as the extent to which the target population comes in contact with a social media–based strategy, Facebook-targeted advertisement (FBTA), designed to connect LCS-eligible individuals in the United States with a digital health communication message. The advertisement served as a digital outreach strategy for promoting engagement with *LungTalk*, an evidence-based intervention aimed at increasing awareness and informed decision-making about LCS.

**Methods:**

As part of the INSPIRE-Lung Study (INnovating Social Media for Prevention: LUNG Cancer Screening Awareness, Knowledge, and Uptake), 5 FBTA campaigns were launched over a 79-day period throughout the United States. Advertisements targeted adults aged 50‐80 years with interests related to smoking or smoking cessation and linked to a study website where participants could complete an eligibility screener and learn more about the trial. Facebook analytics were used to assess *reach*, defined by the number, proportion, and demographic characteristics of individuals exposed to and interacting with FBTA content. Key metrics included total reach, impressions, link clicks, and cost-efficiency.

**Results:**

The FBTA campaigns reached 1,048,191 unique users and generated 3,109,482 impressions (total advertisement displays, including repeat exposures to the same user). A total of 24,816 individuals clicked on the advertisements (2.37% click-through rate), and 7117 completed the eligibility screener. Of those eligible, 1272 (17.9%) met lung screening criteria, and of these, 483 (38% participation rate) enrolled in the trial. The cost per click was US $0.40, and the cost per enrolled participant was US $19.46. Individuals reached via FBTA were demographically diverse and included many who may be disconnected from traditional health care systems.

**Conclusions:**

FBTA is a scalable, cost-effective strategy to achieve population-level *reach* of LCS-eligible adults. By conceptualizing *reach* as exposure to an upstream digital message rather than enrollment alone, this study illustrates how social media can broaden population access to evidence-based cancer prevention tools such as *LungTalk*. Future research should explore embedding intervention content directly into social media platforms and tracking downstream clinical outcomes.

## Introduction

Lung cancer remains the leading cause of cancer-related death in the United States, claiming more lives each year than breast, cervical, colorectal, and prostate cancers combined [1]. Although low-dose computed tomography of the chest is an effective screening tool that can detect lung cancer at early, more treatable stages [2], uptake of lung cancer screening (LCS) remains low, particularly among populations at highest risk [3,4]. Many eligible individuals are unaware that LCS exists or do not know that they meet the criteria, underscoring the urgent need for scalable, population-level communication strategies that increase awareness and engagement [5-7].

The RE-AIM (Reach, Effectiveness, Adoption, Implementation, and Maintenance) framework provides a widely accepted lens for evaluating the population impact of health interventions. Within this framework, *reach* is defined as the absolute number, proportion, and representativeness of individuals who are willing to participate in a given initiative, intervention, or program [8]. However, in digital communication contexts, *reach* should also include those exposed to messaging that serves as a gateway to an intervention, particularly when the goal is to raise awareness and to generate activation toward health care engagement upstream from the health system. Upstream exposure is critical, as prior research demonstrates that initial exposure to preventive health information can subsequently enhance readiness for clinical engagement [9,10].

Social media platforms such as Facebook offer a powerful and cost-effective channel for health communication outreach. Facebook-targeted advertisement (FBTA) enables precise delivery of content to users based on demographic characteristics and behavioral interests, allowing public health practitioners to connect with specific high-risk audiences in real time. Although FBTA has often been used for participant recruitment [11,12], its potential to achieve population-level exposure to health communication interventions has not been fully explored within an implementation science framework.

The objective of this study was to evaluate the *reach* of an FBTA campaign designed to connect LCS-eligible individuals with a digital health communication intervention. Specifically, we examined (1) the number and demographic characteristics of individuals exposed to the FBTA; (2) engagement metrics including click-through rates (CTRs) and progression through the study enrollment pathway; and (3) cost efficiency of FBTA as a population-level outreach strategy. To our knowledge, this is the first study to examine FBTA as a scalable public health outreach strategy for connecting high-risk individuals with a health communication intervention designed to promote awareness of, and informed decision-making about, LCS within an implementation science framework.

## Methods

### Conceptual Framework

This study was guided by the RE-AIM framework, a widely used implementation science model for evaluating the public health impact of interventions delivered in real-world settings [8]. In digital outreach efforts, conceptually, *reach* can be broadened to account for upstream, preintervention exposure that can set the stage for subsequent engagement and trial enrollment [13,14]. Previous studies using digital platforms indicate that broadening the conceptualization of *reach* to include initial digital exposure is important to accurately capture the public health potential of such interventions [15]. In this study, we operationalized *reach* as (1) the number of unique individuals exposed to the FBTA campaign (total reach); (2) the proportion who engaged with the content (CTR); and (3) the demographic representativeness of those exposed compared to the target population for LCS. This operationalization extends the traditional clinic-based conceptualization to include upstream digital exposure as a meaningful public health end point.

This campaign was designed to reach adults likely to meet LCS eligibility criteria by leveraging Facebook’s advertisement targeting features (eg, age, interests, and keywords related to smoking or tobacco use). The ads delivered brief, visually engaging content, with images that served as proxy exposures to the visuals used in the health communication intervention to which participants would later be randomized. For example, some ads featured images of clinical encounters, such as a patient speaking with a clinician or undergoing a lung exam with a stethoscope—images that mirrored scenes from the health communication intervention called *LungTalk* (Table 1). Briefly, *LungTalk* is a computer-tailored health communication and decision support tool designed to educate users about lung health, lung cancer risks, and LCS. The accompanying advertisement text was designed to recruit eligible participants into the study and included statements such as “seeking 50‐80-year-old men and women for online study opportunity about lung health,” and “Are you between 50 and 80 years old and smoke cigarettes or used to smoke? Click to learn more and help improve patient education around lung screening!” Second, we assess user engagement with the FBTA content using Facebook’s built-in analytics to measure visibility (impressions), interaction (click-throughs), and efficiency (cost per click and cost per consent).

**Table 1.** Demographics and Facebook analytics across 5 Facebook-targeted advertisement campaigns for the INSPIRE-Lung Study (INnovating Social Media for Prevention: LUNG Cancer Screening Awareness, Knowledge, and Uptake), a randomized controlled trial to increase lung cancer screening awareness among adults aged 50‐80 years with smoking-related interests in the United States from August 2023 to February 2024 (n=1,048,191 unique users reached). Images in this table were sourced from the Microsoft 365 stock image library and are reproduced under the Microsoft 365 license for stock content for documentation of study materials.

Facebook metrics	Campaign #1	Campaign #2	Campaign #3	Campaign #4	Campaign #5	Overall
Campaign dates	Aug 1‐19, 2023	Sep 4‐15, 2023	Oct 16-Nov 27, 2023	Nov 28, 2023-Jan 28, 2024	Dec 9‐16, 2023	—^a^
Duration, d	19	12	43	62	8	144^b^
Total reach, n (%^c^)	402,817 (38.4)	343,043 (32.7)	75,742 (7.2)	186,544 (17.8)	40,045 (3.8)	1,048,191 (100)
Female	216,513 (53.7)	193,090 (56.3)	55,502 (73.3)	108,804 (58.3)	8175 (20.4)	582,084 (55.5)
Male	180,992 (44.9)	145,921 (42.5)	19,679 (26)	77,162 (41.4)	31,806 (79.4)	455,560 (43.5)
Unknown	5312 (1.3)	4032 (1.2)	560 (0.7)	578 (0.3)	64 (0.2)	10,546 (1.0)
Link clicks, n (%^c^)	7871 (31.7)	5889 (23.7)	7906 (31.9)	2659 (10.7)	491 (2)	24,816 (100)
Female: 45‐54 y	276 (3.5)	267 (4.5)	353 (4.5)	141 (5.3)	22 (4.5)	1059
Female: 55‐64 y	1112 (14.1)	852 (14.5)	1773 (22.4)	471 (17.7)	47 (9.6)	4255
Female: 65+ y	3785 (48.1)	2623 (44.5)	3761 (47.6)	1014 (38.1)	73 (14.9)	11,256
Male: 45‐54 y	132 (1.7)	147 (2.5)	102 (1.3)	118 (4.4)	44 (9)	543
Male: 55‐64 y	505 (6.4)	466 (7.9)	511 (6.5)	333 (12.5)	109 (22.2)	1924
Male: 65+ y	1809 (23)	1476 (25.1)	1319 (16.7)	566 (21.3)	107 (21.8)	5277
Unknown	252 (3.2)	58 (0.98)	87 (1.1)	16 (0.6)	89 (18.1)	502
Impressions, n (%^c^)	751,251 (36.07)	692,496 (33.25)	183,078 (8.79)	361,983 (17.38)	93,682 (4.5)	2,082,490 (100)
Female: 45‐54 y	27,127 (3.6)	27,734 (4)	7216 (3.9)	18,505 (5.1)	4473 (4.8)	85,055
Female: 55‐64 y	98,284 (13.1)	96,094 (13.9)	44,591 (24.4)	73,422 (20.3)	8455 (9)	320,846
Female: 65+ y	285,392 (38)	270,208 (39)	78,966 (43.1)	123,969 (34.2)	12,632 (13.5)	771,166
Male: 45‐54 y	35,629 (4.7)	28,269 (4.1)	2795 (1.5)	20,038 (5.5)	20,339 (21.7)	107,070
Male: 55‐64 y	99,562 (13.3)	81,489 (11.8)	15,279 (8.3)	54,944 (15.2)	27,618 (29.5)	278,892
Male: 65+ y	205,257 (27.3)	188,702 (27.2)	34,231 (18.7)	71,105 (19.6)	20,165 (21.5)	519,460
CTR^d^, %	1.96	1.72	10.44	1.43	1.23	2.37
Cost per click, US $	0.32	0.35	0.38	0.63	1.43	0.40
Total spent, US $	2499.55	2084.82	3000.00	1676.76	701.85	9962.98
Advertisement image	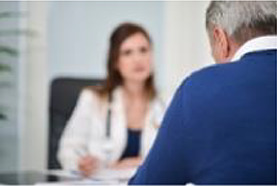	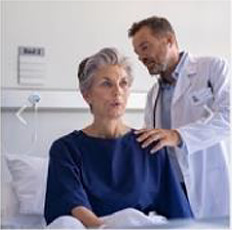	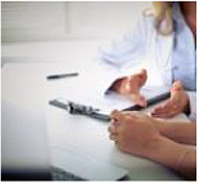	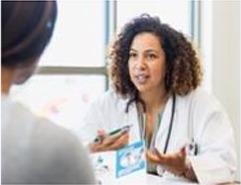	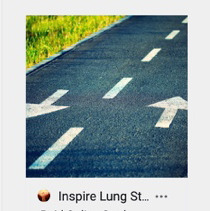	—
Advertisement text	Seeking 50-80-year-old men and women for a study opportunity about lung health.	Are you between 50-80 years old and smoke cigarettes or used to smoke?	Seeking 50-80-year-old men and women to learn more about lung health and screening.	Are you between 50-80 years old and interested in learning more about lung health and screening?	Are you between 50-80 years old and smoke cigarettes or used to smoke?	—

a Not applicable.

b Total days reflect 79 unique recruitment days; some campaigns overlapped (campaign 4 and 5 ran concurrently).

c The percentages in this main row were calculated by using the “Overall” value as the denominator. The percentages for the subcategories were calculated by using the corresponding n values from this row as the denominator.

d CTR: click-through rate (CTR = link clicks/reach ×100).

Most important, FBTA was not the intervention in itself. Rather, it was a communication medium for a study advertisement that enabled scalable outreach to high-risk individuals—adults aged 50‐80 years with a history of smoking—prior to their engagement with the health care system. These individuals represent a population that may have limited engagement with traditional clinical touchpoints (eg, annual physical exam and wellness visits) [16]. Given that LCS awareness remains below 10% nationally [17] and that eligible individuals who smoke are less likely to engage with preventive care [18], FBTA provides an opportunity to deliver targeted health messaging outside traditional clinical encounters—meeting individuals where they already spend time online and potentially catalyzing initial awareness that may precede health care engagement. As such, exposure to the FBTA served as a critical first step in a targeted health communication intervention engagement pathway.

Anchoring this analysis in the RE-AIM framework allows us to expand beyond basic metrics of trial enrollment. Instead, we offer a nuanced understanding of population-level *reach* by quantifying how digital advertising can expand the frontiers of public health communication, particularly for LCS, where knowledge gaps and access barriers persist. While the RE-AIM framework encompasses 5 dimensions, researchers have noted that these components can be examined individually when the research question warrants focused analysis of a specific dimension [14,19]. The present analysis focuses exclusively on *reach* because (1) this paper evaluates the FBTA outreach strategy as a discrete implementation component; and (2) establishing population-level *reach* is a necessary precursor to evaluating downstream dimensions. The remaining RE-AIM dimensions—effectiveness, adoption, implementation, and maintenance—will be evaluated in subsequent publications from the INSPIRE-Lung Study (INnovating Social Media for Prevention: LUNG Cancer Screening Awareness, Knowledge, and Uptake).

### Study Overview

The INSPIRE-Lung Study (ClinicalTrials.gov NCT05824273) is a national, community-based randomized controlled trial designed to increase awareness, knowledge, and uptake of LCS among high-risk individuals [20]. To evaluate reach, we measured exposure to an FBTA campaign that served as a proxy for the health communication intervention.

### Ethical Considerations

This study was approved by the Hackensack Meridian Health Institutional Review Board (Protocol: Pro2022-0860). Informed consent was obtained electronically from all the participants who enrolled in the trial. For aggregated Facebook analytics data (reach, impressions, and clicks), a waiver of informed consent was granted, as these data are anonymized and do not contain personally identifiable information. No personal identifying information was retained from users who only viewed advertisements. All data were deidentified prior to analysis to protect participant privacy and confidentiality. Participants received US $50 for completing baseline and 1-week follow-up surveys and US $25 for completing the 6-month follow-up survey.

### FBTA Campaigns

Between August 2023 and February 2024, 5 discrete FBTA campaigns were used to reach adults aged 50‐80 years in the United States. Campaign durations ranged from 8 to 62 days (Table 1). Campaigns varied in messaging and imagery to test different engagement approaches; however, the primary focus of this analysis is cumulative *reach* rather than comparative campaign effectiveness. Advertisements were developed using Facebook’s advertisement manager platform and tailored to target users based on age, geographic location, and interests related to smoking (eg, cigarettes, vaping, smoking cessation, and nicotine replacement therapy).

Each advertisement linked to the study landing page that briefly described the purpose of the trial and invited users to complete a screening questionnaire to determine LCS eligibility. Although users were not exposed to the full health communication intervention unless they enrolled in the trial, the FBTA messaging was designed to reflect the tone and content focus of health messaging around LCS, emphasizing that screening can save lives and is available to eligible adults with a history of smoking. Thus, FBTA served as a population-level outreach tool and an upstream proxy exposure to the intervention.

Campaigns varied in wording, imagery, and call-to-action phrasing to optimize engagement. Prior to deployment, all ads were reviewed for cultural appropriateness, literacy level, and ethical compliance with best practices for digital recruitment. Real-time monitoring allowed for iterative campaign refinements based on audience response.

### *Reach:* Definition and Metrics

To evaluate *reach* in alignment with the RE-AIM framework, we used both Facebook analytics [21] and internal study data. Facebook analytics [21] provided the real-time performance metrics as follows:

- *Total reach:* The number of unique users who viewed the FBTA content at least once
- *Impressions:* The total number of times the FBTA appeared on users’ screens (which may include multiple exposures per user)
- *Link clicks:* The number of users who clicked the FBTA and were directed to the study website
- *CTR:* The percentage of viewers who clicked on the advertisement
- *Cost metrics:* Cost per click and cost per consented participant

An important consideration is that Facebook’s targeting capabilities allow selection based on age and behavioral interests (eg, smoking-related content) but cannot verify clinical eligibility criteria such as the minimum 20 pack-year smoking history. Therefore, the total reach metric represents exposure among individuals likely but not confirmed to meet screening eligibility. Most importantly, for the purpose of this aim, we conceptualized *reach* as population-level exposure to a public-facing health communication message (delivered via FBTA), independent of whether individuals proceeded to trial enrollment. This approach aligns with RE-AIM’s emphasis on assessing exposure and potential engagement among the broader target population.

### Analytic Strategy

Descriptive statistics were used to summarize campaign-level and cumulative metrics across the 5 FBTA campaigns. We calculated total reach, impressions, CTRs, and cost-efficiency measures. We also examined FBTA impressions by age and sex distribution (Table 1). To assess representativeness of digital engagement, we compared the demographic distribution of link clicks to impressions using chi-square tests. Because Facebook analytics provide aggregate demographic data on impressions rather than unique viewers, we used impression distribution as a proxy for viewer demographics in these comparisons. Additionally, we examined user progression through the digital pathway—from advertisement exposure to click-through to screener completion and trial enrollment—to contextualize how FBTA contributed to downstream engagement with the full health communication intervention.

## Results

Over a 79-day recruitment period between August 2023 and February 2024, 5 US-based FBTA campaigns were launched nationally to reach high-risk individuals eligible for LCS. Across all campaigns, a total of 1,048,191 unique Facebook users were exposed to at least 1 advertisement, representing the total reach of the FBTA strategy. These users generated 3,109,482 total impressions, indicating repeated exposure to the content over time. Among those reached, 24,816 (2.37%) users clicked on the advertisement, which directed them to the study landing page. Of these, 7117 (28.6%) individuals completed the brief eligibility screening questionnaire. A total of 1272 (17.9% of screeners) individuals met LCS eligibility criteria, and of these, 483 (38%) enrolled in the randomized trial. Table 1 details demographic characteristics across the 5 campaigns. Table 2 details the demographic characteristics of participants enrolled in the study.

**Table 2.** Demographic characteristics of participants enrolled in the INSPIRE-Lung Study (INnovating Social Media for Prevention: LUNG Cancer Screening Awareness, Knowledge, and Uptake), a community-based randomized controlled trial to increase lung cancer screening awareness, recruited via Facebook-targeted advertisement in the United States from August 2023 to February 2024 (n=483).

Characteristic	Participants
Age (y), mean (SD)	63.3 (6.7)
Sex, n (%)	
Male	236 (48.9)
Female	246 (50.9)
Nonbinary	1 (0.2)
Race, n (%)	
White	376 (79.3)
Black	70 (14.8)
Asian	7 (1.5)
American Indian	12 (2.5)
Native Hawaiian/Alaskan	2 (0.4)
Multiracial	7 (1.5)
Hispanic, n (%)	
Yes	23 (4.8)
No	454 (95.2)
Education, n (%)	
Less than high school	10 (2.1)
High school graduate	56 (11.6)
Some college	144 (29.9)
College graduate or higher	272 (56.4)
Income, n (%)	
<US $25,000/y	102 (21.2)
$US 25,000-US $50,000/y	141 (29.3)
>US $50,000/y	238 (49.5)
Smoking history (y), mean (SD)	35.5 (10.3)
Currently smokes, n (%)	
Yes	313 (64.8)
No	170 (35.2)

These data demonstrated the substantial *reach* of the FBTA campaign as an upstream population-level exposure strategy. Table 3 presents key campaign metrics across the 5 FBTA iterations. The average cost per link click was US $0.40, and the total campaign cost was US $9962.98, yielding a cost per enrolled participant of US $19.46. Performance varied across campaigns, with Campaign #1 generating the highest total reach (38.4%) and the lowest cost per click (US $0.32). Campaign #3 generated the most link clicks (7906; 31.9%) despite lower total reach. These variations may reflect differences in messaging, imagery, timing, or audience saturation and will be explored in future work.

**Table 3.** Summary of Facebook-targeted advertisement campaign performance metrics for the INSPIRE-Lung Study (INnovating Social Media for Prevention: LUNG Cancer Screening Awareness, Knowledge, and Uptake), a randomized controlled trial targeting lung cancer screening–eligible adults in the United States from August 2023 to February 2024.

Metric	Value^a^
Total reach, number of unique Facebook users	1,048,191
Total impressions, n	3,109,482
Total link clicks, n	24,816
Click-through rate, %	2.36
Screener completers, n	7117
Screen-eligible respondents, n	1272
Enrolled participants, n	483
Total campaign cost, US $	9962.98
Cost per click, US $	0.40
Cost per consent, US $	19.46

a These values are total social media and study metrics.

**Figure 1.**
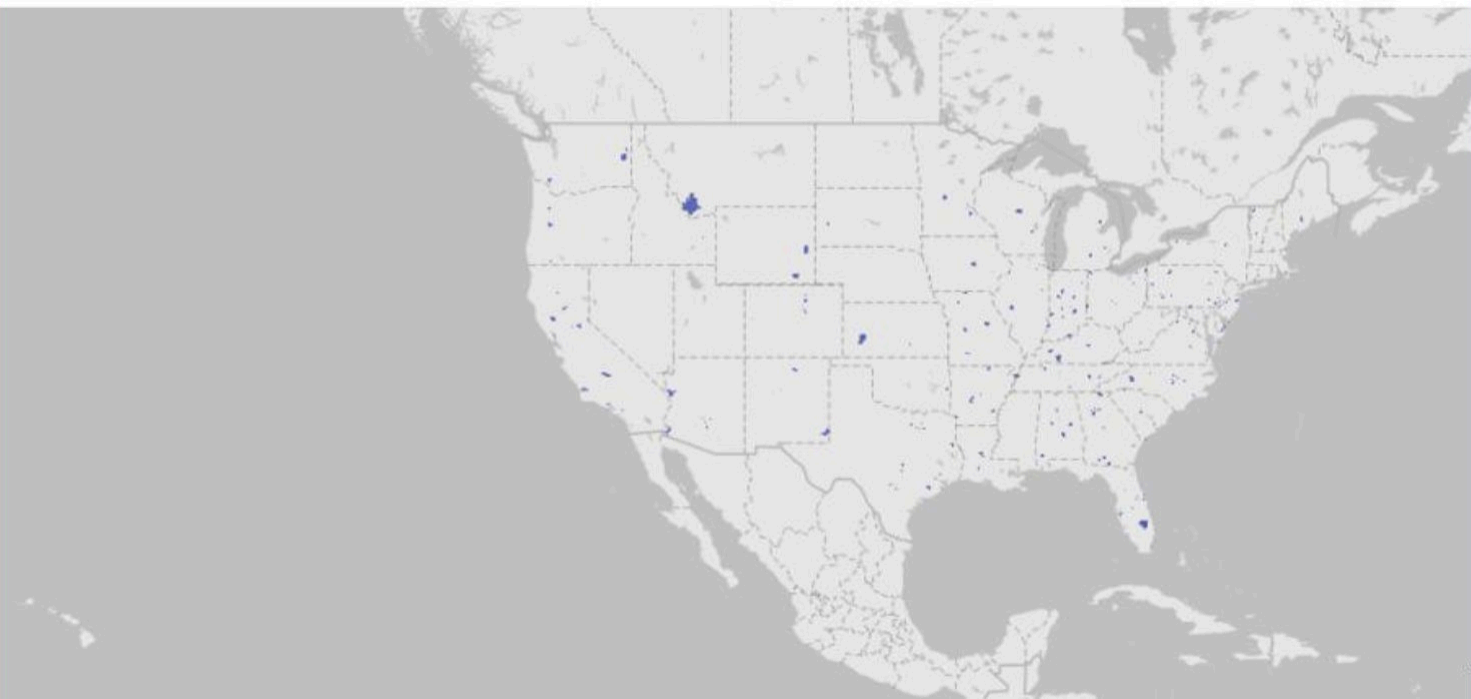
Geographic distribution of INSPIRE-Lung Study participants enrolled via Facebook-targeted advertisement across all 50 US states from August 2023 to February 2024 (n=483). INSPIRE-Lung Study: INnovating Social Media for Prevention: LUNG Cancer Screening Awareness, Knowledge, and Uptake.

Participants reached through the FBTA campaign were geographically and demographically diverse, with representation across all 50 US states (Figure 1). Among those who enrolled, the average age was 63.3 (SD 6.7) years, and 50.9% identified as female participants.

To examine representativeness, we compared the demographic distribution of individuals who clicked on the FBTA to those who viewed the advertisements (using impressions as a proxy for exposure). Chi-square tests revealed significant differences in engagement patterns by age, sex, and geography. Regarding age, individuals aged 65 years and older comprised 61.9% of total impressions but 68% of link clicks, indicating higher engagement among older viewers (*χ*²_2_=436.86; *P*<.001). CTRs increased with age: 0.83% for ages 45‐54 years, 1.03% for ages 55‐64 years, and 1.28% for ages 65+ years. Regarding sex, female participants comprised 56.5% of impressions but 68.2% of link clicks, demonstrating significantly higher engagement compared to male participants (*χ*²_1_=1337.74; *P*<.001). The CTR for female participants (1.41%) was notably higher than for male participants (0.86%). Geographic distribution also differed significantly across US Census regions (*χ*²_3_=26.89; *P*<.001), with the South accounting for the highest proportion of both impressions (36.2%) and clicks (37.1%).

However, even among individuals who did not proceed to enrollment, exposure to the FBTA message about LCS represents a meaningful first step toward increasing awareness and consideration of screening. This broader lens on *reach* captures the use of digital messaging strategies upstream from health care system engagement.

## Discussion

### Principal Findings

This study demonstrates the feasibility and use of FBTA as a digital strategy to achieve broad, population-level *reach* to LCS-eligible individuals using tailored health messaging. Guided by the RE-AIM framework [8], we operationalized *reach* not as trial enrollment but as exposure to a communication message that served as an upstream proxy for a health communication intervention. Across 5 campaigns in the United States, we reached over 1 million unique individuals within the target age range and smoking history risk profile for LCS. These findings highlight the potential of FBTA to serve as a scalable public health outreach tool that promotes engagement with evidence-based communication interventions.

Most importantly, this analysis distinguishes between *exposure* and *enrollment*. While a subset of users exposed to the FBTA ultimately enrolled in the trial and received the *LungTalk* intervention or control condition depending on randomization, the majority did not progress beyond the initial digital touchpoint. Nonetheless, this first contact with a tailored screening message represents a meaningful public health outcome, particularly in a context where lack of awareness is a persistent barrier to LCS uptake. Although FBTA effectively reached individuals who may not have engaged with traditional health care outreach, the extent to which initial digital exposure translates into sustained clinical behavior change remains uncertain and warrants further longitudinal investigation. In addition, given the fact that LCS awareness is so low in general (<10%) [17], increasing population level awareness regardless of screening eligibility is of great value. In line with RE-AIM, conceptualizing *reach* in this broader way captures the real-world potential of digital tools to connect individuals with preventive health information upstream of clinical interaction. The remaining RE-AIM dimensions—effectiveness, adoption, implementation, and maintenance—will be evaluated in subsequent publications from the INSPIRE-Lung Study. Specifically, effectiveness will be assessed through the primary RCT outcomes examining *LungTalk’s* impact on LCS awareness, knowledge, and screening uptake. Adoption and implementation metrics will examine health care system and provider engagement, while maintenance will assess sustained behavior change at 6- and 12-month follow-ups.

To better understand the representativeness of the FBTA campaigns, we compared the demographics of enrolled participants with the screening population in the American College of Radiology’s Lung Cancer Screening Registry (ACR LCSR), as reported by Silvestri et al [22]. In that study of over 1 million individuals screened for lung cancer with low-dose computed tomography between 2015 and 2019, the population was predominantly White (91.6%), with only 7.4% Black and 0.9% Asian individuals among those with known race/ethnicity data. In contrast, our FBTA-recruited sample was notably more diverse: 79.3% White, 14.8% Black (double the ACR LCSR proportion), and 4.8% Hispanic. This suggests that FBTA may be particularly effective at reaching racial and ethnic minority populations who have historically been underrepresented in clinic-based LCS.

Additional demographic patterns also differed from the ACR LCSR. While the registry population skewed older adults (42.6% aged 65+ y) and female participants (approximately 54%), our enrolled participants showed a more balanced sex distribution (50.9% female participants, 48.9% male participants) and a mean age of 63.3 (SD 6.7) years. The ACR LCSR also reported that 61.4% of screened individuals currently smoked; similarly, 64.8% of our enrolled participants currently smoked, indicating that FBTA successfully reached individuals with active smoking behaviors who represent a priority population for LCS outreach. Notably, Black and Hispanic individuals demonstrated lower adherence to annual screening in the ACR LCSR, suggesting that alternative outreach strategies like FBTA may help address these disparities at the initial awareness and engagement stage.

However, certain groups, such as those with lower educational attainment or without internet access, may still be underrepresented in digital outreach, underscoring the importance of multichannel approaches. In our sample, 56.4% had a college degree or higher, which exceeds national averages and suggests potential selection bias toward more educated individuals.

The FBTA strategy also proved highly cost-efficient. While the average cost per link click was US $0.40 and the cost per enrolled participant was US $19.46, the cost per view (or impression) was substantially lower, at just over US $0.003 per exposure. This underscores the potential of FBTA to deliver high-volume, targeted health communication at minimal per-person cost. Our cost per enrolled participant (US $19.46) compares favorably to published estimates for traditional recruitment methods in cancer prevention research. Community-based recruitment strategies have reported costs ranging from US $50 to over US $500 per participant, depending on intensity and population [23,24]. Direct mail campaigns for health research typically cost US $100‐200 per enrolled participant [25]. Our prior work using FBTA for LCS research demonstrated similar cost efficiencies [11]. Compared to traditional outreach strategies such as print mailings, community events, telephone outreach, or provider referrals, which often require substantial personnel time and logistical coordination, FBTA offers real-time precision targeting and scalability. This is especially advantageous for reaching populations who may have limited engagement with the health care system. According to the Pew Research Center (2024), approximately 68% of the US adults use Facebook, with relatively consistent usage across income levels and geographic regions [26]. Most importantly, Facebook penetration among adults aged 50‐64 (70%) years and 65+ (50%) years approaches or mirrors general population estimates, suggesting potential to reach individuals across the socioeconomic spectrum. While Facebook users as a whole may not be systematically disconnected from health care, our targeting strategy—focusing on individuals with smoking-related interests—may identify a subpopulation with elevated barriers to care, as prior research indicates that individuals who currently smoke are less likely to have a usual source of care and less likely to receive preventive services [18]. Moreover, the low rate of LCS uptake nationally (<6%) suggests substantial numbers of eligible individuals are not engaging with screening through traditional clinical pathways. FBTA may be particularly valuable for reaching individuals living in rural areas, those without consistent primary care access, and those less likely to encounter LCS information through conventional clinical channels.

### Limitations

Nevertheless, several limitations should be acknowledged. First, while FBTA allowed for targeted delivery of content based on self-reported profile characteristics and behavioral interests, we could not verify screening eligibility (eg, 20+ packs per year smoking history) until individuals clicked through to the screening questionnaire. As such, Facebook’s *reach* metric represents exposure among individuals likely but not confirmed to meet screening eligibility criteria and may include a significant proportion who are not personally eligible for LCS. The fact that only 17.9% of those who completed the screener met eligibility criteria illustrates this gap between interest-based targeting and clinical eligibility. However, we conceptualize this exposure as meaningful from a public health perspective: raising awareness about LCS, even among some individuals who may not personally meet eligibility criteria, has value, as these individuals may share information with eligible family members or friends. Given the inherently social nature of Facebook, this kind of peer-sharing and diffusion of information could amplify the campaign’s impact within networks of individuals with shared interests in smoking or cessation.

Second, although the study provides valuable insights into digital *reach*, it does not assess the quality or depth of engagement with the FBTA message among those who viewed but did not interact with the advertisement. Future studies could incorporate passive analytics (eg, time spent viewing or scrolling behavior) or embed brief *LungTalk*-derived content within the advertisement itself to assess this dimension.

Third, there are broader ethical and equity considerations when leveraging commercial social media platforms for health communication. While Facebook offers powerful precision-targeting capabilities, Facebook algorithms may inherently exclude or underrepresent certain demographic groups, potentially biasing exposure toward users already digitally active or engaged, which could inadvertently widen health disparities [27]. Ensuring equitable digital engagement will be essential as these strategies are scaled [27]. Future research should systematically monitor and adjust targeting criteria to mitigate this risk.

Fourth, we cannot definitively rule out exposure to fake or bot accounts, though Facebook’s platform integrity measures are designed to minimize such accounts. Additionally, our downstream metrics (screener completion, eligibility verification, and trial enrollment) require human engagement that automated accounts cannot replicate, providing confidence that our enrollment figures represent genuine participants.

Despite these limitations, our findings have important implications for cancer prevention and public health communication. Reaching high-risk individuals who are not yet engaged with the health care system remains a major barrier to improving LCS rates. FBTA offers a powerful, scalable method to extend the reach of evidence-based interventions like *LungTalk* and to promote awareness among populations traditionally missed by clinic-based strategies.

Future efforts should explore how social media–based outreach can be integrated with other implementation strategies, such as patient navigation or community partnerships, to support screening uptake. Additionally, embedding the intervention content itself directly into the FBTA (rather than linking out) will allow for even greater scalability and immediacy of impact.

### Next Steps and Future Directions

Building on the demonstrated feasibility and population-level *reach* of FBTA to connect with LCS-eligible individuals, several strategic opportunities emerge for advancing digital public health communication and implementation efforts.

First, future research should explore embedding core components of the intervention, such as tailored *LungTalk* messaging or *LungTalk* itself, directly within the FBTA interface. This would allow for immediate engagement with decision support content at the moment of exposure, reducing drop-off and increasing the likelihood that viewers absorb key information, even if they do not proceed to enrollment. Embedding brief video clips, testimonials, or tailored visual messages adapted from *LungTalk* could help bridge the gap between exposure and education. For example, a randomized comparative effectiveness study could assess whether embedding *LungTalk* intervention components directly within FBTA enhances immediate knowledge gains compared to ads linking externally to a study website.

Second, optimizing message design and delivery remains critical. Future campaigns may consider using systematic A or B testing of message framing, imagery, tone, and calls to action, with attention to behavioral science principles and cultural relevance. Tailoring content for priority populations, such as Black, Hispanic or Latino, or rural adults with elevated lung cancer risk, can further improve engagement and equity.

Third, future studies should examine how digital *reach* translates into downstream behavioral and clinical outcomes. For example, FBTA-driven exposure could be linked to subsequent engagement with patient navigation, telehealth consults, or direct referral systems to LCS programs. Implementation strategies that combine digital outreach with systems-level linkage to care hold promise for closing the gap between awareness and screening uptake.

Fourth, collaboration across outreach and communication sectors will be essential for scaling impact. Partnering with health systems, public health departments, advocacy organizations, and community-based coalitions can support broader dissemination, localized adaptation, and sustainable infrastructure for ongoing FBTA campaigns.

Finally, ensuring equity in digital health communication is imperative. As social media becomes an increasingly common tool for public health messaging, attention must be paid to disparities in internet access, digital literacy, and algorithmic exposure. Mixed methods evaluations, community-engaged research, and monitoring of campaign delivery across demographic groups can help identify and address gaps. Most importantly, even users who are not personally eligible for LCS may serve as information conduits within their social networks, amplifying campaign effects through peer sharing and expanding awareness among others who may have missed the original exposure.

In addition, FBTA offers a scalable, adaptable, effective, and cost-efficient platform for delivering tailored health communication content upstream of clinical care. By refining and integrating this strategy with broader implementation efforts, future research and practice can more effectively promote equitable access to LCS information and ultimately reduce disparities in early detection and outcomes.

### Conclusions

This study demonstrates the potential of FBTA to achieve meaningful population-level *reach* to individuals eligible for LCS, as defined by the RE-AIM framework. By delivering a tailored communication message that served as a proxy exposure to a health communication intervention that promotes awareness and informed decision-making about LCS, FBTA enabled contact with over 1 million adults within the eligible age range, many of whom are unlikely to encounter such information through traditional health care pathways.

By broadening *reach* to encompass initial exposure to health-promoting digital messages, this analysis highlights the importance of upstream engagement in implementation science and public health practice. FBTA proved to be a cost-efficient and scalable outreach strategy capable of penetrating social networks, initiating awareness, and opening pathways for subsequent engagement with LCS services.

As public health increasingly embraces digital platforms to extend its reach, integrating social media strategies with evidence-based interventions offers a promising direction for reducing disparities in screening and early detection. Future efforts should build on this foundation by embedding intervention content directly into advertisement formats, testing links to downstream care, and ensuring equitable access across diverse communities.

## Abbreviations

ACR LCSR: American College of Radiology’s Lung Cancer Screening Registry
CTR: click-through rate
FBTA: Facebook targeted advertisement
INSPIRE-Lung Study: INnovating Social Media for Prevention: LUNG Cancer Screening Awareness, Knowledge, and Uptake
LCS: lung cancer screening
RE-AIM: Reach, Effectiveness, Adoption, Implementation, and Maintenance
