# Correction: Leveraging Social Media to Achieve Population-Level Reach of Lung Cancer Screening-Eligible Individuals: A RE-AIM Framework Perspective

**DOI:** 10.2196/94664

**Published:** 2026-03-05

**Authors:** Lisa Carter-Bawa, Jamie S Ostroff, Susan M Rawl, Erin A Hirsch, Smita C Banerjee, Andrew Ciupek, Robert Skipworth Comer, Minal Kale, Katherine T Leopold, Patrick O Monahan, James E Slaven Jr, Francis Valenzona, Renda Soylemez Wiener, Ana Guadalupe Vielma

**Affiliations:** 1 Cancer Prevention Precision Control Institute, Center for Discovery & Innovation, Hackensack Meridian Health, 123 Metro Blvd, 6th Floor, 6400 Pod, Nutley, NJ, 07110, United States, 1 646-246-2118; 2 Cancer Prevention & Control Program, Georgetown Lombardi Comprehensive Cancer Center, Washington, DC, United States; 3 Department of Psychiatry & Behavioral Science, Memorial Sloan Kettering Cancer Center, New York, NY, United States; 4 School of Nursing, Indiana University, Indianapolis, United States; 5 Go2 for Lung Cancer, Washington, United States; 6 School of Informatics, Indiana University, Indianapolis, United States; 7 Department of Medicine, Internal Medicine, Icahn School of Medicine at Mount Sinai, New York, United States; 8 Hackensack Meridian School of Medicine, Hackensack Meridian Health, Nutley, United States; 9 Department of Biostatistics and Health Data Science, Indiana University School of Medicine, Indianapolis, IN, United States; 10 Center for Health Optimization & Implementation Research, VA Boston Healthcare System, Boston, MA, United States; 11 National Center for Lung Cancer Screening, Veterans Health Administration, Washington, DC, United States; 12 The Pulmonary Center, Boston University Chobanian & Avedesian School of Medicine, Boston, MA, United States

In “Leveraging Social Media to Achieve Population-Level Reach of Lung Cancer Screening-Eligible Individuals: A RE-AIM Framework Perspective” [1], the authors noted one error.

The title previously appeared as:

Leveraging Social Media to Achieve Population-Level Reach of Lung Cancer Screening–Eligible Individuals: RE-AIM Framework–Based Secondary Analysis of a Randomized Controlled Trial

The title now reads:

Leveraging Social Media to Achieve Population-Level Reach of Lung Cancer Screening-Eligible Individuals: A RE-AIM Framework Perspective

The correction will appear in the online version of the paper on the JMIR Publications website, together with the publication of this correction notice. Because this was made after submission to PubMed, PubMed Central, and other full-text repositories, the corrected article has also been resubmitted to those repositories.
